# Renal denervation reverses ventricular structural and functional remodeling in failing rabbit hearts

**DOI:** 10.1038/s41598-023-35954-3

**Published:** 2023-05-29

**Authors:** Ting-Chun Huang, Li-Wei Lo, Yu-Hui Chou, Wei-Lun Lin, Shih-Lin Chang, Yenn-Jiang Lin, Shin-Huei Liu, Wen-Han Cheng, Ping-Yen Liu, Shih-Ann Chen

**Affiliations:** 1 Division of Cardiology, Department of Medicine, Taipei Veterans General Hospital, No. 201, Sec. 2, Shi-Pai Road, 11217 Taipei, Taiwan; 2 Institute of Clinical Medicine, College of Medicine, National Cheng Kung University, Tainan, Taiwan; 3 Institute of Clinical Medicine, and Cardiovascular Research Institute, National Yang-Ming Chiao-Tung University, Taipei, Taiwan; 4 Division of Cardiology, Department of Internal Medicine, National Cheng Kung University Hospital, College of Medicine, National Cheng Kung University, Tainan, Taiwan; 5 Cardiovascular Center, Taichung Veterans General Hospital, Taichung, Taiwan; 6 National Chung Hsing University, Taichung, Taiwan

**Keywords:** Cardiovascular biology, Neuroscience

## Abstract

Renal denervation (RDN) suppresses the activity of the renin–angiotensin–aldosterone system and inflammatory cytokines, leading to the prevention of cardiac remodeling. Limited studies have reported the effects of renal denervation on ventricular electrophysiology. We aimed to use optical mapping to evaluate the effect of RDN on ventricular structural and electrical remodeling in a tachycardia-induced cardiomyopathy rabbit model. Eighteen rabbits were randomized into 4 groups: sham control group (*n* = 5), renal denervation group receiving RDN (*n* = 5), heart failure group receiving rapid ventricular pacing for 1 month (*n* = 4), and RDN-heart failure group (*n* = 4). Rabbit hearts were harvested for optical mapping. Different cycle lengths were paced (400, 300, 250, 200, and 150 ms), and the results were analyzed. In optical mapping, the heart failure group had a significantly slower epicardial ventricular conduction velocity than the other three groups. The RDN-heart failure, sham control, and RDN groups had similar velocities. We then analyzed the 80% action potential duration at different pacing cycle lengths, which showed a shorter action potential duration as cycle length decreased (*P* for trend < 0.01), which was consistent across all groups. The heart failure group had a significantly longer action potential duration than the sham control and RDN groups. Action potential duration was shorter in the RDN-heart failure group than the heart failure group (*P* < 0.05). Reduction of conduction velocity and prolongation of action potential duration are significant hallmarks of heart failure, and RDN reverses these remodeling processes.

## Introduction

Heart failure (HF) caused by insults impairing ventricular systolic or diastolic function results in poor end-organ perfusion and is a leading cause of death^1^. The renin–angiotensin–aldosterone system, sympathetic nervous system, and several cytokines are activated in HF patients to maintain cardiac output and tissue perfusion^2^. The overactivity of the sympathetic nervous system plays a central role in the vicious cycle in a failing heart^3^.

Renal denervation (RDN) decreases renal and systemic sympathetic outflows through the cardiorenal axis^4^. Although RDN in the SYMPLICITY HTN-3 trial did not significantly lower systolic blood pressure compared to sham treatment^5^, the SPYRAL HTN-OFF MED trial found significant effects of lowering office and 24-h ambulatory blood pressures by RDN^6^. Beyond resistant hypertension, some clinical studies or case reports have suggested that RDN might have the ability to treat various cardiovascular diseases, including atrial fibrillation^7^, refractory ventricular tachycardia^8,9^ and HF^10,11^. In humans, the benefits of reversing atrial structural remodeling, identified by echocardiography^12^ and electroanatomical mapping^13^, could be the mechanism. Tsai et al. provided proof of histopathological and functional remodeling of the stellate ganglion after RDN by using a canine model^14^. Our previous studies showed the antifibrotic effect of RDN on rabbit models^15,16^. The changes in ventricular electrophysiological characteristics after RDN are unclear.

In this study, we aimed to investigate the possible reverse remodeling effects of RDN on left ventricle function and electrophysiological properties in a tachycardia-induced cardiomyopathy rabbit model.

## Results

### Echocardiographic data

**Figure 1:**
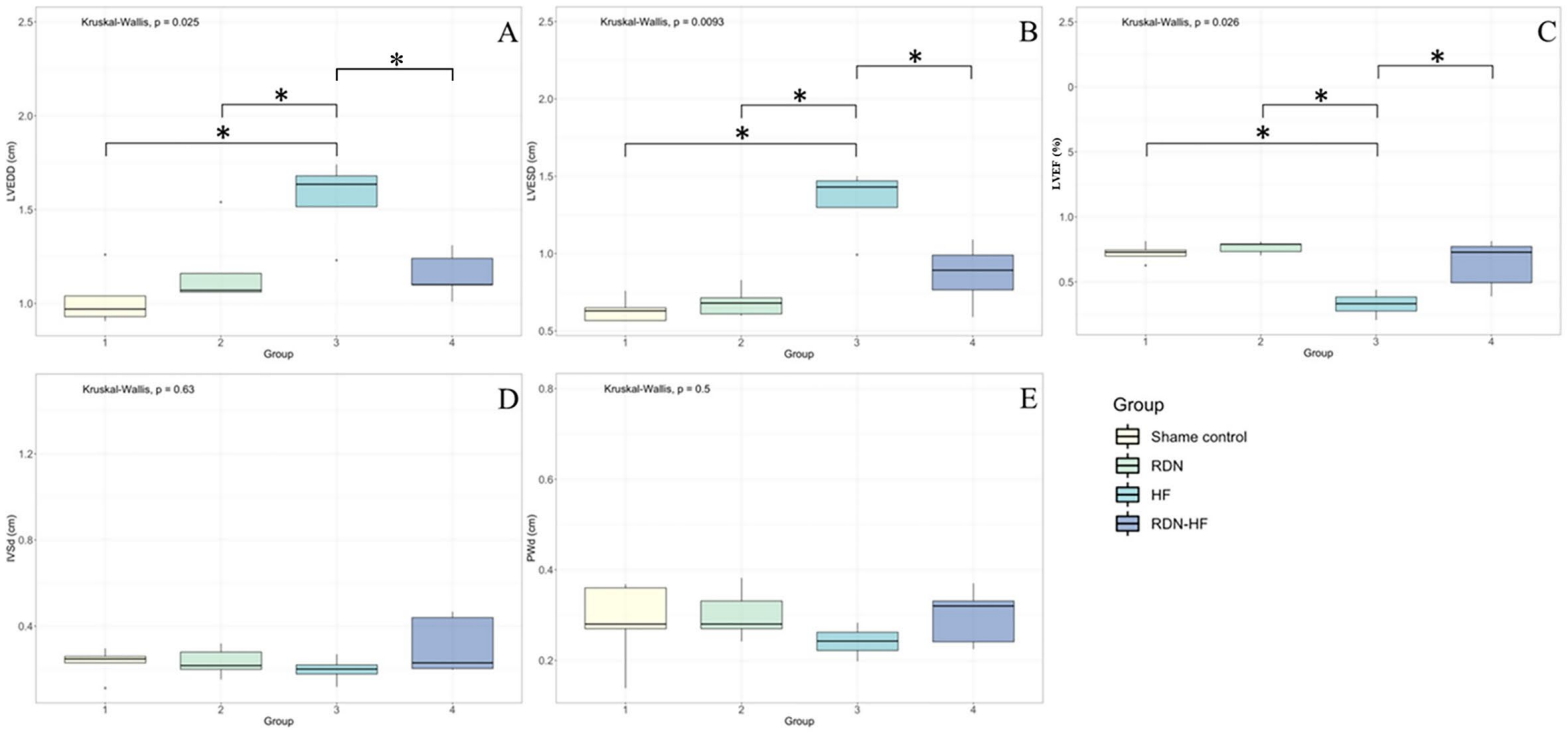
Echocardiographic measurements. Echocardiographic parameters measured from 4 groups are presented. * *p* < 0.05 between two groups, Dunn’s post hoc test. LVEDD—left ventricular end-diastolic diameter; LVESD—left ventricular end-systolic diameter; LVEF—left ventricular ejection fraction; IVSd—interventricular septum diameter; PWd—posterior wall diameter.

The Fig. 1 shows the results of LV ejection fraction, systolic and diastolic LV diameters, and interventricular and posterior wall thicknesses. After 4 weeks of continuous pacing, the HF group had significantly dilated end-systolic and end-diastolic LV diameters compared with the other three groups, and the LV end-diastolic diameters in the HF group were significantly larger than those in the sham control and RDN groups. No significant change in LV wall thickness, including the interventricular septum and posterior wall, after continuous pacing in the HF group was found. The RDN-HF group had no differences in chamber diameter or LV ejection fraction compared with the sham control and RDN groups.

### Ventricular epicardial conduction velocity

**Figure 2:**
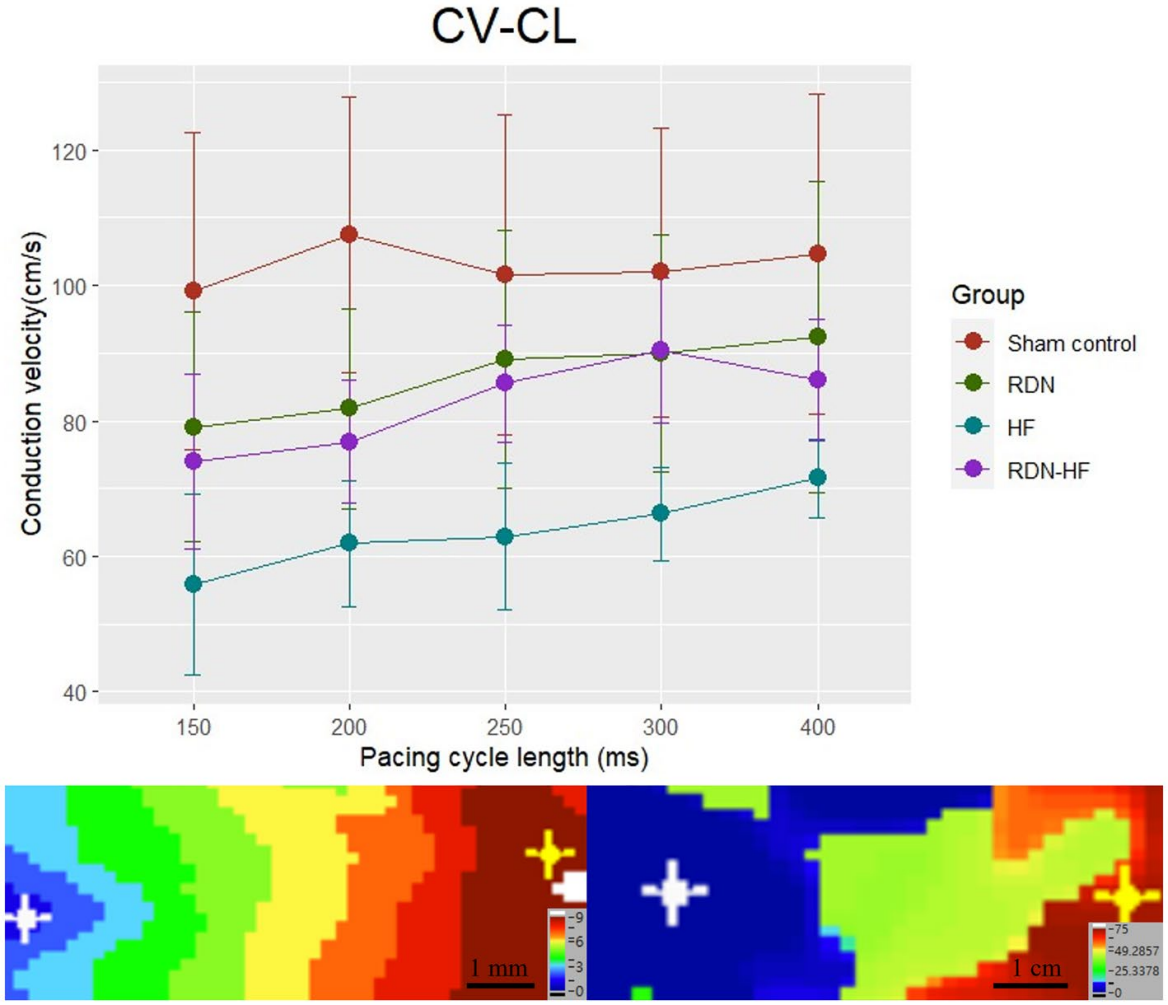
(**A**) Conduction velocity (cm/s). The mean CV with one standard deviation at different pacing CLs in each group is presented. The epicardial CV in the HF group was significantly slower than that in the other three groups. (**B**) At the pacing CL of 300 ms, an isochrone map of the HF-RDN rabbit. (**C**) At the CL of 300 ms, an isochrone map of the HF rabbit. CV—conduction velocity; CL—cycle length.

The conduction velocities (CVs) at different pacing cycle lengths (CLs) in each group are presented in Fig. 2 and Table 1. Epicardial CVs in the RDN-HF group gradually slowed as pacing CLs became slower (*p* for trend < 0.05). This phenomenon was not found in the other groups. The HF group had a significantly slower epicardial CV than the sham control at each pacing CL. The HF group had significantly slower CVs at pacing CLs of 250 to 300 ms than the RDN and RDN-HF groups (*p* < 0.05). No differences were found between the sham control, RDN, and HF-RDN groups.

**Table 1:** CV (cm/s) at different pacing CLs in each group.

Cycle length	Sham control (mean ± SD, N = 5)	RDN group (mean ± SD, N = 5)	HF group (mean ± SD, N = 4)	RDN-HF group (mean ± SD, N = 4)	*p* value
400 ms	104.518 ± 23.628	92.342 ± 22.880	71.554 ± 5.793*	86.001 ± 8.987	0.002
300 ms	101.927 ± 21.314	90.030 ± 17.484	66.254 ± 6.897^+^	90.339 ± 10.701	0.001
250 ms	101.652 ± 23.617	89.024 ± 18.976	62.899 ± 10.836^+^	85.481 ± 8.728	< 0.001
200 ms	107.396 ± 20.360	81.768 ± 14.673	61.893 ± 9.332*	76.894 ± 9.123	< 0.001
150 ms	99.153 ± 23.467	79.082 ± 16.906	55.818 ± 13.304*	73.958 ± 12.899	0.018
*p* for Trend	0.959	0.437	0.067	0.046	

*HF* heart failure; *RDN* renal denervation; *RDN-HF* renal denervation-heart failure; *SD* standard deviation.

*p < 0.05, HF group versus sham control group.

+ *p* < 0.05, HF group versus sham control, RDN, or RDN-HF group.

### Ventricular action potential duration and action potential restitution

**Figure 3:**
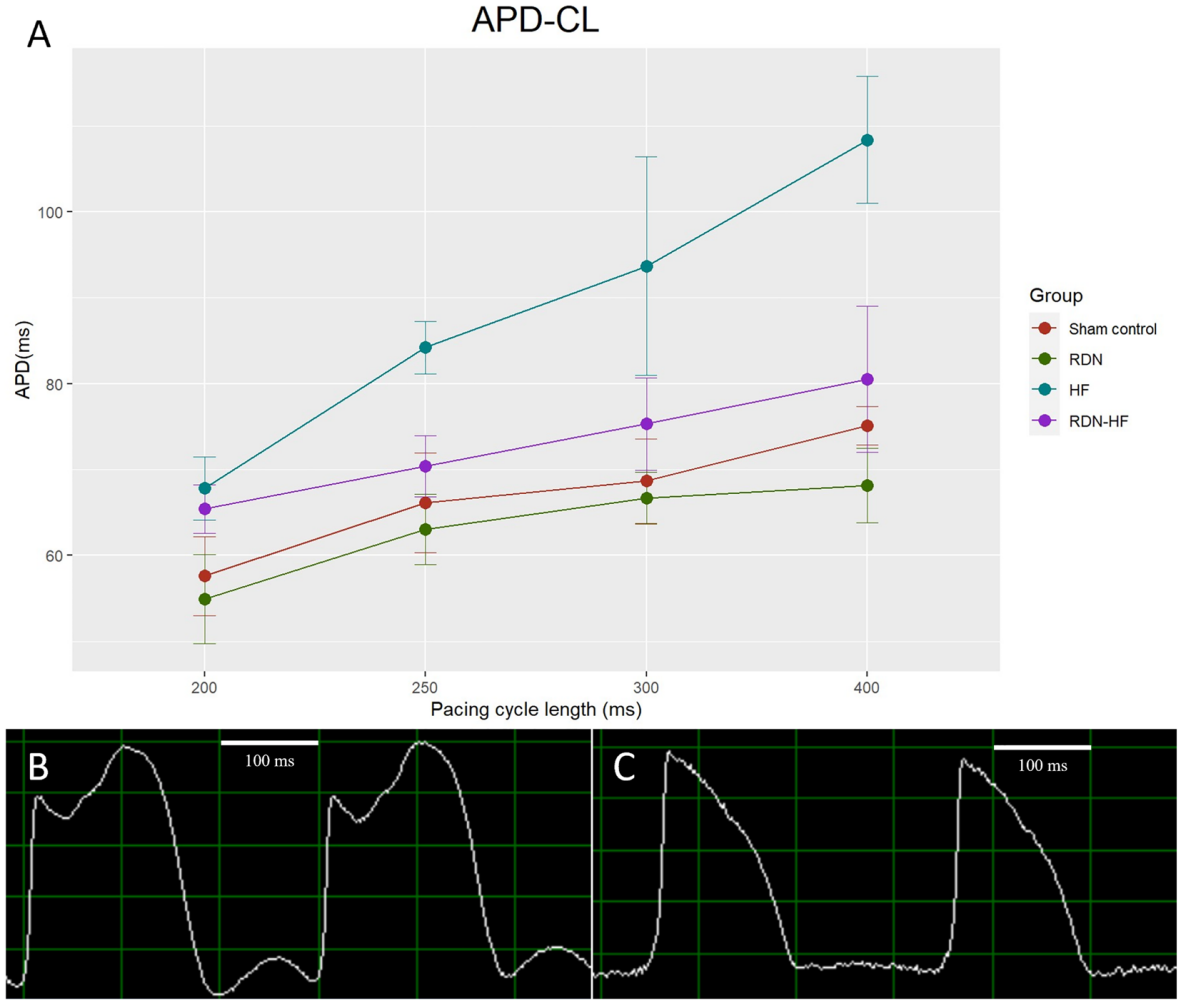
Action potential duration (ms). (**A**) The mean action potential duration with one standard deviation at different pacing CLs in each group is presented. The HF group had a significantly longer APD than the other groups. (**B**) Action potential signals at the pacing CL of 300 ms in the HF rabbit. (**C**) Action potential signals at the pacing CL of 300 ms in the RDN rabbit. APD—action potential duration; CL—cycle length.

The Fig. 3 and Table 2 show the 80% action potential duration (APD_80_) at pacing CLs of 400 ms, 300 ms, 250 ms and 200 ms in each group. APD_80_ had a steady decrease as pacing CLs became shorter in each group (*p* for trend < 0.05). At a pacing CL of 400 ms, the RDN group had a shorter APD_80_ than the other three groups. This phenomenon was not found at the pacing CL of 300, 250, or 200 ms. The RDN group had a significantly shorter APD_80_ than the RDN-HF group at all pacing CLs. The HF group had a significantly longer APD_80_ than the sham control and RDN groups at all pacing CLs. The RDN-HF group had a significantly shorter APD_80_ than the HF group.

**Table 2:** APD_80_ at different pacing CLs in each group.

Cycle length	Sham control (mean ± SD, N = 5)	RDN group (mean ± SD, N = 5)	HF group (mean ± SD, N = 4)	RDN-HF group (mean ± SD, N = 4)	*p* value
400 ms	75.107 ± 2.222^+^	68.116 ± 4.336^$^	108.362 ± 7.372*	80.541 ± 8.510^#^	< 0.001
300 ms	68.662 ± 4.926	66.672 ± 3.024^$^	93.684 ± 12.701*	75.334 ± 5.372	< 0.001
250 ms	66.111 ± 5.786	63.050 ± 4.124^$^	84.198 ± 3.077*	70.374 ± 3.564^#^	< 0.001
200 ms	57.608 ± 4.613	54.932 ± 5.163^$^	67.809 ± 3.683*	65.420 ± 2.819	< 0.001
*p* for trend	< 0.001	< 0.001	< 0.001	< 0.001	

*HF* heart failure; *RDN* renal denervation; *RDN-HF* renal denervation-heart failure; *SD* standard deviation.

^+^*p* < 0.05, RDN versus sham.

^$^*p* < 0.05, RDN versus RDN-HF.

*p < 0.05, HF versus sham control or RDN.

^#^*p* < 0.05, HF versus RDN-HF.

^@^*p* < 0.05, sham versus RDN-HF.

**Figure 4:**
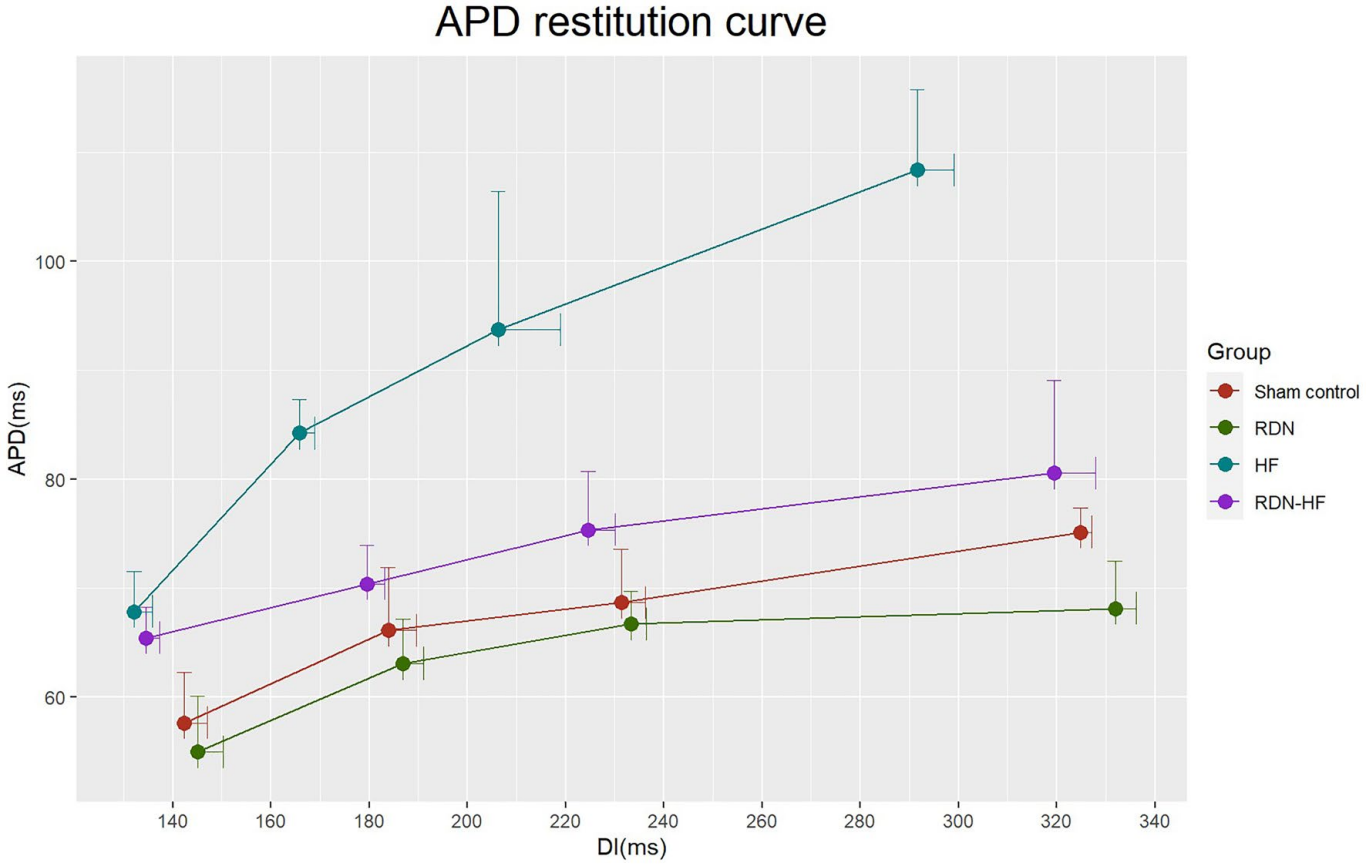
APD restitution curve. (**A**) steeper slope was noted in the HF group. In contrast, the RDN-HF group maintained a flat slope of the APD restitution curve, similar to the sham control and RDN groups. APD—action potential duration; DI—diastolic interval.

The action potential duration restitution curve is presented in Fig. 4. Although all slopes were less than 1, the HF group had the steepest slope (0.487), and the RDN-HF group, like the sham control and RDN groups, had a flat slope in each part of the APD restitution curve.

## Discussion

### Main findings

In this study, rapid ventricular pacing-induced HF resulted in ventricular structural remodeling, including chamber dilatation and worsening of ventricular systolic function, in line with our previous studies. These changes demonstrated the consistency of pacing-induced HF models. This study also found HF-related electrophysiological remodeling, including slower CV, longer APD_80_, and steeper slope of the APD restitution curve. RDN itself and possible procedure-related complications did not result in any significant change in heart function, chamber size, or conduction properties. The only difference between the HF and RDN-HF groups was RDN. The slight but nonsignificant impairments in left ventricular ejection fraction, chamber dilatation, and decreased conduction velocity in the RDN-HF group suggest the potential for prevention of LV structural and electrical remodeling by RDN.

### RDN prevents structural remodeling of a failing heart

Chronic sustained tachycardias result in a ventricular dysfunction that is a reversible process, and this concept is demonstrated by both animal models and human studies that have shown improved ventricular function after terminating arrhythmia or rate control^17–19^. Various degrees of ventricular dysfunction in animal models depend on the rate and duration of pacing^20^. Chronic rapid ventricular pacing animal models show markedly dilated LV end-systolic and end-diastolic chambers with wall thinning^21^ or preservation of wall thickness^22^. In our study, the HF group had a more dilated end-systolic LV chamber than the other three groups and a more dilated end-diastolic LV chamber than the sham control and RDN groups. In contrast, echocardiography showed no significant changes in LV systolic or diastolic chamber size in the RDN-HF group. The HF group had lower LV systolic function after long-term ventricular pacing, but the RDN-HF group had limited tachycardia-related systolic dysfunction.

### RDN prevents functional remodeling of a failing heart

Our study also showed that the RDN-HF group had a slower CV as the pacing CL shortened (*p* for trend < 0.05), but this phenomenon was not found in the other groups. These data suggested that dynamic changes in CV occur more prominently in advanced stages of remodeling^23^. The HF group had significantly slower CVs than the sham control at all pacing CLs and the RDN and RDN-HF groups at pacing CLs of 250 to 300 ms, but prevention of the decrease in CV was found in the RDN-HF group. This might be related to lesser sympathetic nerve stimulation, which is thought to increase the CV^24,25^.

In normal hearts, the shortening of APD as heart rate increases is essential^26^, and our study found that this phenomenon existed consistently in advanced heart remodeling. APD prolongation and enhanced dispersion of refractoriness are hallmarks of repolarization remodeling in the failing heart^27^. Our experiment yielded similar findings: a marked prolongation of APD_80_ and a steeper slope of the APD restitution curve in the HF group compared with the sham control group and RDN group. The RDN-HF group had a significantly shorter APD than the HF group, which suggested that RDN prevented the prolongation of APD_80_ in failing hearts. The RDN-HF group had a significantly longer APD than the RDN group, a finding that provides biological proof that our pacing animal model resulted in cardiac remodeling.

### RDN prevents arrhythmogenesis

RDN reduces the occurrence of ventricular arrhythmias and attenuates the rise in LV end-diastolic pressure during ischemic insults in a swine model^28^, and decreases the QT interval, the dispersion of the QT interval, and the ventricular effective refractory period in a canine model^29^. Our group reported that RDN prevents structural remodeling through its antifibrotic effects^15^. In this study, we demonstrated RDN effects on electrophysiological characteristics, including preventing the slowing of CV, prolonging APD_80_, and maintaining a flat slope of the APD restitution curve in the failing myocardium. These factors are related to a decrease in the peak density of transient sodium currents in cardiomyocytes isolated from the failing heart^30^ and downregulation of connexin-43 mRNA and protein in end-stage ischemic and dilated cardiomyopathy^31^, and could potentially be arrhythmogenic by promoting unidirectional conduction block and re-entrant arrhythmias^32^.

Recent case series^8,9^ and case reports^33,34^ have discussed the potential benefits of RDN in the treatment of ventricular tachycardia in ischemic and nonischemic cardiomyopathy. Similarly, limited clinical evidence has shown beneficial effects when RDN is applied to patients with chronic HF^11^. Future clinical studies may focus on patients with mild to moderate hypertension and HF to test the biological concept that RDN reduces HF progression, ventricular arrhythmia attack, and appropriate implantable cardioverter defibrillator therapy in short-term and long-term follow-up. In this study, we only performed experiments on male rabbits. Sex differences in APD and QT interval in humans and other animal species, including rabbits, have been reported^35^. Sex differences in the potential beneficial effects of RDN could be another issue worth studying.

### Limitations

First, we performed the RDN procedure before programmed rapid ventricular pacing, which means that systemic and renal sympathetic activations decreased in the early stage of disease progression. It is unknown whether RDN still works in the very late stage of HF. Second, raping pacing-related motion artifacts and rapid pacing-induced ventricular fibrillation, especially in the HF group, prohibited us from doing the analysis correctly at a pacing cycle length of 150 ms. Third, we did not assess the activation of the sympathetic system directly by measuring sympathetic nerve activity or norepinephrine spill-over rate before harvesting.

## Methods

### Animal preparation

The present study protocol was reviewed and approved by the Institutional Animal Care and Committee of Taipei Veterans General Hospital (IACUC2014-087) and was in strict accordance with the US National Institutes of Health or European Commission guidelines. All surgical procedures were performed under general anesthesia, and all efforts were made to minimize animal suffering.

In this study, we included a total of 18 male New Zealand white rabbits, age 12 weeks, weight 2.0–3.0 kg, from Shu-Lin Breeding facility, New Taipei, Taiwan. Each animal was housed in its own cage (530 × 630 × 320 mm). All rabbits were kept in a temperature- (22–25 °C) and humidity-controlled (50–70%) environment with a 12-h light–dark cycle (lights on from 7:00 am to 7:00 pm). All rabbits had unlimited access to food (Laboratory Rabbit Diet 5326 HF, PMI, Richmond, IN) and water.

### Experimental procedures

Eighteen rabbits were randomized to 4 groups. Group 1 was the sham-operated control group (control group, *n* = 5). Group 2 underwent the RDN procedure only (RDN group, *n* = 5). Group 3 was the HF model, which underwent pacemaker implantation to induce HF (HF group, *n* = 4). Group 4 was the HF model given RDN (RDN-HF group, *n* = 4).

### Creation of the rabbit HF model

HF was induced in the rabbits by rapid right ventricular pacing through programmable left ventricular (LV) pacemakers (Sigma SS303; Medtronic, Minneapolis, MN, USA), implanted as described previously^36,37^. In brief, an intramuscular injection of a mixture of Zoletil (10 mg/kg) and xylazine (5 mg/kg) was given at the beginning of the procedure. Repeated injections of Zoletil and xylazine were administered as required to maintain a deep level of anesthesia. An intravenous catheter was inserted into the marginal ear vein for anesthesia. The rabbits were then intubated and ventilated artificially with room air or supplemented with oxygen to maintain oxygen saturation ≥ 95%. After opening the left third intercostal space, part of the pericardium was cut to expose the posterior surface of the heart. Two unipolar pacing leads (pacing lead 6491; Medtronic, Ltd., Minneapolis, MN) fixed to the LV posterior free wall were connected to the dual-chamber pacemaker, which was implanted subcutaneously at the left subaxillary area. After the procedure, the rabbits received an infusion of antibiotic (gentamicin, 5–8 mg/kg) and ibuprofen (2–10 mg/kg) in their water for 3 days for pain relief.

One week after the procedure, a pacemaker programmer, CareLink™ Programmer (Medtronic, Minneapolis, MN, USA), enabled with wireless telemetry, was used to check the sensitivity and pacing threshold of both pacing leads, and then the pacemaker was set to DOO mode to achieve ventricular pacing of 300 beats per minute in the experimental groups. Surface and intracardiac electrocardiograms were monitored by the surface electrocardiography and pacemaker programmer once per week to adjust the pacing to the maximum ventricular rate, thus allowing for 1:1 capture. At the end of a 4-week pacing, HF status was confirmed by the presentation of signs of ascites, edema, drowsiness and dyspnea, which were compatible with experimental and clinical HF ^38^.

### RDN procedure

Rabbits were fasted for one night before the procedure, and bilateral chemical and surgical RDNs were performed by using the same anesthesia method described above. We approached the bilateral kidneys through a mid-abdominal incision. Surgical RDN was performed by cutting all visible nerves around the bilateral renal hilus, followed by an RDN chemical delivered in 20% phenol solution for 10 to 15 min at approximately 1 cm of the adventitia from the renal artery. After the RDN procedures, animals were observed for re-equilibration for 1 h, and the abdomen was closed layer by layer with suture after checking for bleeding. If rabbits had undergone a pacemaker implantation, we performed RDN 7 days after that procedure.

### Echocardiography

We evaluated cardiac function right before the optical mapping procedure with an ultrasound system (CX50 xMATRIX; Koninklijke Philips N.V., Eindhoven, The Netherlands). After a uniform anesthetic protocol, we shaved the anterior chest and upper abdomen hair and placed the rabbit in the right lateral decubitus position. LV end-diastolic diameter, LV end-systolic diameter, interventricular septum diastolic thickness, and LV posterior wall diastolic thickness were measured using M mode in the parasternal long-axis view. LV ejection fraction was calculated with the Teichholz formula. All measurements were obtained from 3 cardiac cycles, and the data were averaged.

### Optical mapping protocol

Four weeks after the RDN procedure and/or ventricular rapid pacing, the epicardial ventricular activation pattern was studied by using a cardiac optical mapping system as described^39^. Continuous ventricular pacing by the pacemaker was terminated before harvesting the rabbit heart. After hanging on the Langendorff perfusion system, the rabbit hearts were stained with RH 237 (5 mg/mL solution in dimethyl sulfoxide, Invitrogen™) and then perfused with blebbistatin (1 mg/mL solution in dimethyl sulfoxide, Tocris Bioscience), a myosin inhibitor, to minimize cardiac motion-related artifacts. The hearts were excited with 4 light-emitting diode modules, and the signals were gathered at 2-ms sampling intervals for 1000 frames, acquired from 128 × 128 sites simultaneously over a 30 × 30 mm^2^ area in each aspect of the heart. For each optical recording, data were acquired continuously for 2 s.

**Figure 5:**
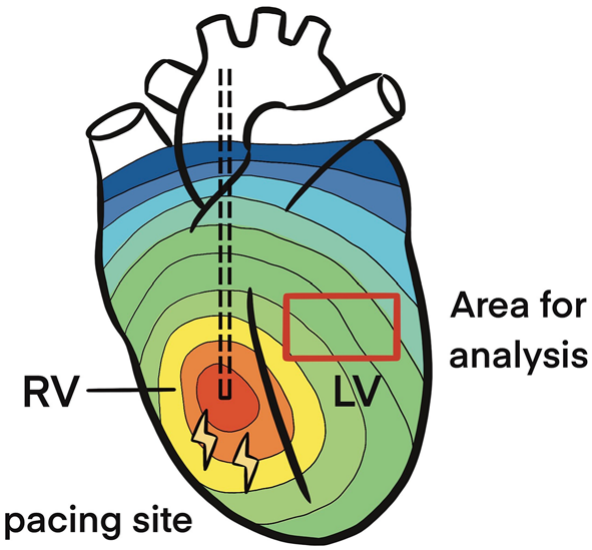
Illustration of the experiment.

Three unipolar needle electrodes were inserted into the LV myocardium to detect the cardiac rhythm, and a bipolar electrode was inserted into the right ventricular apex for continuous pacing. Programmed electrical stimulation with different CLs (400, 300, 250, 200, and 150 ms) was performed with an electrophysiologic stimulator (Bloom DTU-215B, Fischer Medical, Wheat Ridge, USA) that delivered a constant-current pulse width of 3-ms duration. During continuous right ventricular pacing, rectangles at the center of the anterior free wall of the LV (Fig. 5) were used to evaluate CV. While calculating CV, a single vector method that measured the difference in activation times and the distance between two selected points along the apparent longitudinal axis was used. In the same area, the average action potential duration (APD) of three consecutive heart beats was measured. The APD was measured from the steepest deflection of the slope of phase 0 to the time of 80% repolarization, which was denoted as APD_80_, at pacing CLs of 400, 300, 250, and 200 ms. The diastolic interval (DI) was calculated as CL minus APD_80_.

### Statistical analysis

Categorical variables are presented as frequencies and percentages, whereas continuous variables are reported as means and standard deviations. Continuous variables were preliminarily compared by the Kruskal‒Wallis test (https://CRAN.R-project.org/package=PMCMRplus). If *p* was < 0.05, Dunn’s test was used to run pairwise comparisons. Trend analysis in the general linear model is reported. All statistical tests were 2-sided, and a *p* value less than 0.05 was considered statistically significant. All analyses were performed with statistical software R, version 3.6.3 for Windows.

### Ethics approval and consent to participate

The present study protocol was reviewed and approved by the Institutional Animal Care and Committee of Taipei Veterans General Hospital (IACUC2014-087) and was in strict accordance with the US National Institutes of Health or European Commission guidelines. In addition, this study complied with ARRIVE, and the details are presented in the ARRIVE Essential 10 author checklist.

## Conclusion

Structure and electrophysiological remodeling are important hallmarks of failing hearts. RDN potentially benefits failing hearts by minimizing these remodeling processes. Further clinical trials using next-generation RDN devices are needed to provide more biological evidence.

## Data Availability

All data generated or analyzed during this study are included in this published article.

## Abbreviations

APD: Action potential duration
CL: Cycle length
CV: Conduction velocity
HF: Heart failure
LV: Left ventricle
RDN: Renal denervation
